# Flow-Mediated Dilation in Healthy Young Individuals Is Impaired after a Single Resistance Exercise Session

**DOI:** 10.3390/ijerph17145194

**Published:** 2020-07-18

**Authors:** Gustavo Vieira de Oliveira, Elisaldo Mendes Cordeiro, Mônica Volino-Souza, Cristina Rezende, Carlos Adam Conte-Junior, Thiago Silveira Alvares

**Affiliations:** 1 Nutrition and Exercise Metabolism Research Group, Nutrition Institute, Federal University of Rio de Janeiro, Macaé, Rio de Janeiro 27971-525, Brazil; gvo.vieira@gmail.com (G.V.d.O.); prof.elisaldo@gmail.com (E.M.C.); mvolinosouza@gmail.com (M.V.-S.); cristinarzd@gmail.com (C.R.); carlosconte@hotmail.com (C.A.C.-J.); 2 Postgraduate Program in Bioactive Products and Biosciences, Federal University of Rio de Janeiro, Macaé, Rio de Janeiro 27971-525, Brazil; 3 Postgraduate Program in Food Science, Chemistry Institute, Federal University of Rio de Janeiro, Rio de Janeiro 21941-909, Brazil

**Keywords:** FMD, endothelial function, vascular responsiveness, strength training

## Abstract

The current pool of data investigating the effects of a single resistance exercise session on endothelial function is divergent and inconclusive. Therefore, the purpose of the present study was to evaluate the effect of a single resistance exercise session on flow-mediated dilation (FMD) in trained individuals. Eleven healthy, young, recreationally resistance-trained individuals participated in the study. After determining the resistance exercise workload, the participants performed three sets of 10–12 repetition of leg press and leg extension exercises. By using ultrasound equipment, brachial artery FMD was assessed before (PRE) and 30 min after (POST) the resistance exercise protocol or resting (control) to evaluate endothelial function. A significant reduction in FMD response (PRE: 5.73% ± 1.21% vs. POST: 4.03% ± 1.94%, *p* < 0.01) after resistance exercise was observed, accompanied by a large effect size (*d* = 1.05). No significant difference was observed in FMD in the control condition (PRE: 5.82% ± 1.19% vs. POST: 5.66% ± 1.24%, *p* = 0.704). Additionally, no significant difference in baseline brachial artery diameter between resistance exercise (PRE: 3.30 ± 0.32 vs. POST: 3.40 ± 0.34 mm, *p* = 0.494) and resting (PRE: 3.64 ± 0.41 vs. POST: 3.67 ± 0.62 mm, *p* = 0.825) was observed. Our findings showed that a single resistance exercise session induced a reduction in FMD in resistance-trained individuals.

## 1. Introduction

Impairment of endothelial function is the first step toward a cardiovascular event [1]. Endothelial function can be assessed by ultrasound, which detects changes in the diameter of conduit arteries from baseline measurements to postocclusion diameter, induced by applying the vascular occlusion test (VOT) [1,2]. Previous studies [2,3,4] demonstrated that blood flow velocity in the brachial artery increases after inducing a five minutes of ischemia with a cuff placed over the forearm, thereby generating a frictional force on the artery wall (shear stress). The shear stress stimulates the vascular endothelium to release nitric oxide (NO), which modulates vascular tonus by means of relaxation of smooth muscle cells lining the arteries (vasodilation) [2]. Therefore, the assessment of flow-mediated dilation (FMD) by ultrasound represents a NO-mediated, endothelium-dependent vasodilator response, providing important information on endothelial function in conduit arteries [3,5].

It is well known that short-term (e.g., 8 weeks) physical exercise training improves FMD (endothelial function) and possibly decreases cardiovascular risk [6]. Physical exercise improves vascular function through a variety of hemodynamics stimuli, including increasing shear stress, arterial blood pressure, and intramuscular pressure caused by rhythmic muscle contraction during exercise [7,8]. Although the mechanisms controlling the positive effects of physical exercise on vascular function are not entirely understood, it is likely that increased blood flow to the muscles during exercise, and consequently shear stress, may modulate artery structure and/or function by means of NO release [9,10]. A previous study showed improvements in FMD after eight weeks of an exercise training program without changes in cardiovascular risk factors, reinforcing the premise that hemodynamic stimuli caused by physical training play crucial roles in modulating endothelial function [6]. In light of this, Pedralli et al. [11] recently reported that either resistance exercise or aerobic exercise, including the combination of aerobic and resistance exercise, for eight weeks improved FMD in individuals with prehypertension or hypertension. Similarly, Olson et al. [12] also reported that 12 months of resistance training improved FMD in overweight women. Despite the abundance of evidence regarding the positive effects of long-term resistance training on the FMD response [11,12] studies showed increased [13,14,15] or reduced FMD [16,17] after a single aerobic and resistance exercise session. Therefore, current evidence regarding the impact of a single physical exercise session on endothelial function is conflicting and the underlying mechanisms are poorly understood.

Resistance exercise programs involve several confounding factors, including intensity, volume, and cadence of exercise, among others, all of which may elicit different FMD responses after acute resistance exercise [10]. Previous studies investigated the effect of light- to moderate-intensity (performing approximately 12–24 repetitions near maximal exertion) resistance exercise on the FMD response in trained individuals. Given that the intensity of exercise seems to be determinant in modulating the FMD response [10,17], the present study sought to investigate whether performing a single high-intensity resistance exercise session (performing 60–70 maximal repetitions) reduced FMD in healthy young individuals. Investigating the effect of a single resistance exercise session on FMD may be advantageous in terms of prescribing appropriate exercise protocols and experimentally controlling confounding variables (e.g., intensity, volume, interval rest between sets). Therefore, it was hypothesized that a single resistance exercise session would decrease FMD after a single resistance exercise session.

## 2. Materials and Methods

### 2.1. Participants

Eleven individuals were invited to participate in the present study via announcement in flyers and poster. Baseline characteristics of the participants are shown in Table 1. All participants were young and healthy and engaged in a resistance training program for at least 6 months. Exclusion criteria included taking antioxidants or preworkout supplements, as well as anabolic steroid usage for at least 6 months prior to beginning experimental procedures. Volunteers were also excluded if they had any osteomioarticular injuries that could impair lower limb exercise. Premenopausal women were studied within the first 5 days of their menstrual cycle, since endothelial function measurements of the brachial artery are documented to fluctuate with menstrual phase [1,2]. All experimental procedures were performed after explaining the nature of the study and obtaining written consent from participants. All procedures of the study were performed in accordance with the ethical standards of the declaration of Helsinki and approved by the institutional ethics committee of the Federal University of Rio de Janeiro, Brazil (protocol number: 53392216.0.0000.5699).

### 2.2. Experimental Procedures

**Figure 1:**
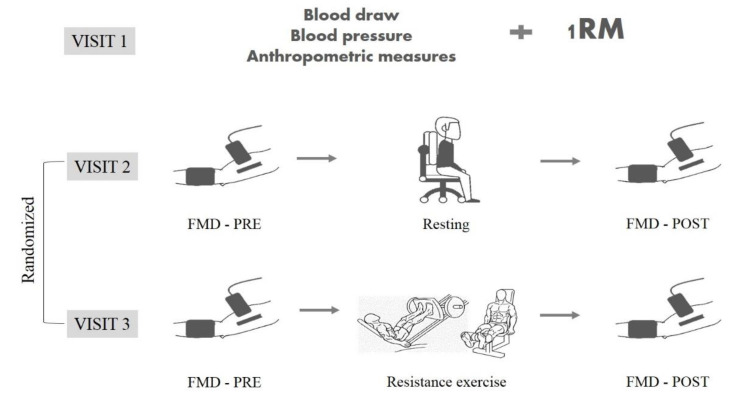
Experimental design. RM = repetition maximum; FMD = flow-mediated dilation.

All experimental procedures took place on three occasions. The first visit to the laboratory involved anthropometric measures, blood drawing, blood pressure measurement and one repetition maximum (1-RM) test for leg press (Movement^®^, São Paulo, Brazil) and leg extension (Movement^®^, São Paulo, Brazil) exercises. The 1-RM test was estimated according to a previous study [18]. The second and third visits involved the assessment of FMD at baseline (PRE) and 30 min after (POST) the resistance exercise protocol or a resting control condition (no exercise) (Figure 1). The second and third visits were randomized in a balanced way (1:1). There was a 1-week interval between each visit at the laboratory. All visits were held between 08:00 and 11:00 am in an airconditioned room (constant temperature of 22–25 °C). In order to avoid any potential interference on FMD measurement in the present study, the participants were instructed to fast for at least 8 h. All participants restricted physical exercise and caffeine consumption two days before each visit.

### 2.3. Resistance Exercise Protocol

After arriving in the laboratory, the participants warmed up at the leg press machine by performing 15 repetitions at 50% 1-RM. Afterward, the volunteers performed the leg press exercise, followed by the knee extension exercise. The range of motion was from 0° to approximately 90° of knee flexion for the leg press exercise and from 90° to 180° for the leg extension exercise. The cadence of the exercise was controlled using a metronome sound (1/1 sec concentric/eccentric phase). All participants performed three sets of 10–12 RM of the leg press and leg extension exercises. Concentric failure was defined when the participants could not complete another repetition with strict biomechanics (changing movement patterns). The interval rest between each exercise set, including the warm-up, was 2 min. A single exercise session lasted approximately 20 min. At the end of each set, subjects were instructed to continue to breathe regularly in order to avoid abnormal elevation of blood pressure.

### 2.4. Flow-Mediated Dilation Assessment

Flow-mediated dilation measurement was conducted in accordance with previous studies [2,19]. FMD was assessed using an ultrasound approach with a high-resolution linear array transducer (13 MHz), coupled with computer-assisted analysis software (e-TRACKING system, AlokaCo., Tokyo, Japan). With this software, changes in the brachial artery could be assessed in real-time through an automated edge detection system. Longitudinal images of the brachial artery were obtained with the transducer fixed on the medial aspect of the dominant arm, approximately 2 cm above the medial epicondyle of the humerus. The transducer was fixed on the arm by a special arm-holding device (MIST-100, Saraya Co., Osaka, Japan). The brachial artery diameter was acquired as per a 30 s baseline measure, after which a cuff was placed around forearm (3 cm above wrist) and inflated to 250 mm Hg for 5 min. After cuff deflation, the brachial artery diameter was continuously monitored for 2–4 min to detect the peak artery diameter. FMD was determined as the percentage change in diameter from baseline to peak arterial diameter. The coefficient of variation was intraday 5.1% ± 0.31% and interday 5.1% ± 0.12%. The coefficient of variation was determined based on the nine healthy young subjects, who were evaluated by the same trained examiner.

### 2.5. Statistical Analysis

To detect statistical difference in the FMD measurement at PRE and POST resistance exercise and control (no exercise) a two-way ANOVA test (condition × time) was performed. Moreover, ANOVA was also used to identify a potential gender effect among the participants. The magnitude (effect size) of the resistance exercise was calculated by Cohen’s d, with values <0.2 considered trivial, 0.2 to 0.5 a small effect, 0.5 to 0.8 a moderate effect, and ≥0.8 a large effect. Statistical significance was set at a *p* value of <0.05 and the results were expressed as means ± standard deviation (SD). A commercially available statistical package (IBM SPSS Statistics version 22 for Mac, Armonk, NY, USA) was used for statistical analysis. The graphics were designed using GraphPad Prism 5 (GraphPad Software, San Diego, CA, USA).

## 3. Results

**Figure 2:**
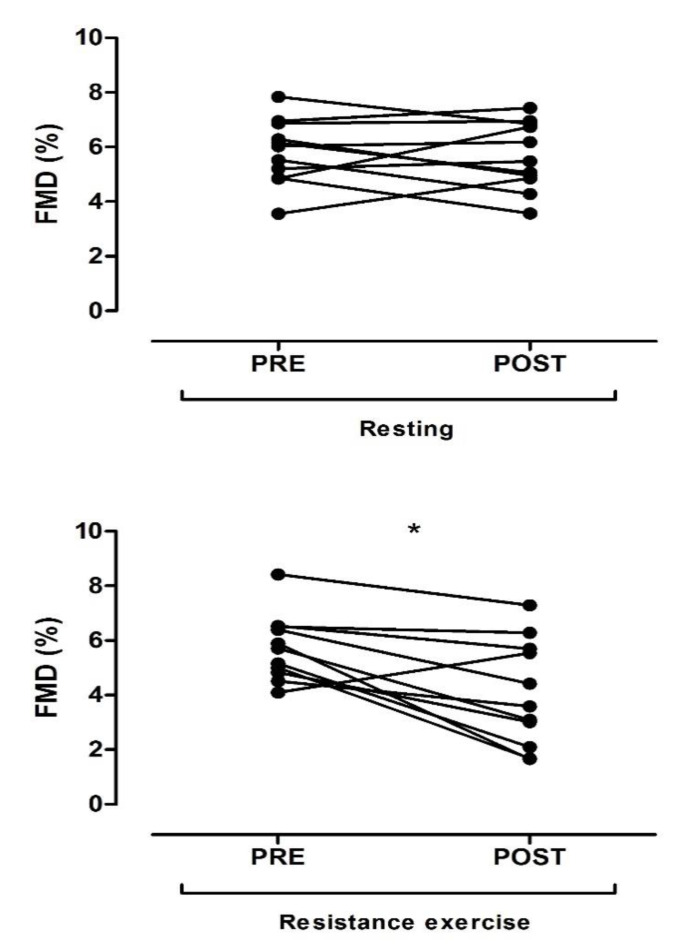
Flow-mediated dilation (FMD) before (PRE) and after (POST) resistance training or resting. * Significantly different from PRE (resistance training).

Baseline characteristics of the participants are shown in Table 1. Figure 2 shows the FMD response assessed at PRE and POST resistance exercise and resting (no exercise).

There was a significant main effect regarding time (*p* = 0.005) and an interaction effect (*p* = 0.017) regarding time per condition (exercise or resting). Post hoc testing revealed a significant reduction in FMD after resistance exercise (PRE: 5.73% ± 1.21% vs. POST exercise: 4.03% ± 1.94%, *p* = 0.001). Additionally, a larger effect size (*d* = 1.05) was observed. No significant difference in FMD in the control condition (PRE: 5.82% ± 1.19% vs. POST resting 5.66% ± 1.24%, *p* = 0.704), followed by a small effect size (*d* = 0.13), was observed. Additionally, FMD was significantly lower (*p* = 0.029) after exercise (POST: 4.03% ± 1.94%) compared to resting (POST: 5.66% ± 1.24%). Moreover, there was no significant interaction effect between the condition (exercise or resting) and gender (*p* = 0.582).

There was no significant difference observed in the main effect regarding time of baseline brachial artery diameter during resistance exercise (PRE: 3.30 ± 0.32 mm vs. POST: 3.40 ± 0.34 mm, *p* = 0.494) and resting (PRE: 3.64 ± 0.41 mm vs. POST: 3.67 ± 0.62 mm, *p* = 0.825). Further, no significant difference was observed in the main effect regarding time for peak brachial artery diameter during resistance training (PRE: 3.48 ± 0.33 mm vs. POST: 3.52 ± 0.32 mm, *p* = 0.817) and resting (PRE: 3.85 ± 0.40 mm vs. POST: 4.02 ± 1.08 mm, *p* = 0.497).

## 4. Discussion

The present study aimed to assess the brachial artery FMD response in resistance-trained individuals after a single resistance exercise session. Our findings demonstrated a significant reduction in FMD assessed at 30 min after resistance exercise. In agreement with our findings, Gonzales et al. [17] reported that brachial artery FMD decreased in recreationally active individuals after intermittent slow contraction handgrip exercise (30 min of exercise with short periods of rest). The authors reported that longer periods of time spent in muscle contraction during slow contraction strongly contributed to increased blood pressure, which is related to FMD impairment [17]. Some possible explanation for reduced FMD after a single resistance exercise session may be related to the release of endothelin-1 (a vasoconstrictor molecule) and increased blood pressure [20,21,22].

In contrast to our findings, other studies showed a significant increase in FMD [14,15]. Varady et al. [14] demonstrated an increase in brachial artery FMD response in resistance-trained individuals after three sets of 8–12 repetitions performed near maximal exertion using a leg press machine. Phillips et al. [15] also reported improvements in brachial artery FMD assessed at 30 min after two or three sets of 6–8 repetitions (near maximal exertion) performed using a leg press machine. Indeed, the lack of consistent findings should not come as a surprise, since resistance exercise involves many variables (intensity, volume, interval rest between sets, movement cadence, etc.), all of which may influence the FMD response [10]. Although it is difficult to control all resistance exercise variables, the number of repetitions performed (i.e., the volume of training) may play an important role in the FMD response. For example, the participants in the present study underwent six sets of 10–12 maximal repetitions, totaling approximately 60–70 repetitions in the resistance training session, whereas the participants from the study by Phillips et al. performed approximately 12–24 repetitions near maximal exertion [15]. Furthermore, it is important to mention that in the present study, all participants performed maximal repetitions, which is one way to increase the intensity of exercise. Conversely, in the studies by Varady et al. and Phillips et al. [14,15], the participants did not perform maximal repetitions during resistance exercise. One strength of the current study was that the participants performed both higher intensity (maximal repetition) and volume (number of repetitions) of resistance exercise than previous studies [14,15], which may explain the reduced FMD after exercise [10,17]. The intensity of exercise elicits increases in blood pressure, which is associated with the release of vasoconstrictor molecules (endothelin-1) and reduced FMD after exercise [20,21,22].

Regardless of these divergent findings with respect to the acute effect of exercise on FMD in healthy resistance-trained individuals, long-term resistance exercise is a well-established way to improve FMD in individuals at cardiovascular disease (CVD) risk, including those with prehypertension or hypertension [11] and overweight women [12]. Therefore, in no way should the practice of resistance exercise be seen as a health-damaging exercise model, but the acute effect of maximal resistance exercise on FMD could be taken into consideration during resistance exercise prescription for individuals that possess lower FMD values, such as those with CVD risk factors [19]. In a clinical perspective, considering that resistance exercise programs are widely recommended worldwide, the effect of acute exercise on the FMD response should be controlled (by adjusting volume and/or intensity of exercise), particularly in individuals at high risk for CVD. Additionally, the effect of acute resistance exercise to reduce FMD may last approximately two hours after a single physical exercise session, followed by a return of FMD to baseline values (transient reduction of FMD) [23,24]. Therefore, the acute effect of resistance exercise on FMD should be interpreted with caution, without assuming it to be a basal/permanent phenomenon.

### Experimental Consideration

A limitation of this study was the lack of blood pressure and blood flow velocity assessment during the resistance exercise protocol. It was mentioned that the mechanism by which exercise-induced high blood pressure decreases FMD is associated with high levels of shear stress (a frictional force exerted by blood flow velocity on endothelium) during prolonged periods, which may negatively affect nitric oxide bioavailability [22]. However, Gonzales et al. [17] observed that a reduction in FMD after resistance exercise was associated with higher blood pressure, but not with shear stress. Thus, although it is well established that blood flow velocity and blood pressure during resistance exercise both increase [7,25], future studies assessing such hemodynamics parameters are recommended in order to provide more information on this topic. Moreover, it is noteworthy that Gonzales et al. [17] observed a reduction in FMD of 3.3% (from 6.9% to 3.6%) after handgrip exercise, whereas a reduction in FMD of 1.7% (from 5.7% to 4.0%) was reported in this study. However, in Gonzales et al.’s study, the participants performed handgrip exercises (upper limb exercises), whereas in the current study, the participants performed leg press and leg extension exercises (lower limb exercises), with FMD being assessed in the upper limb (brachial artery) in both studies. This suggests that the decline in FMD after exercise may be influenced by the location where FMD is being assessed (i.e., exercising vs. nonexercising limb). It is also important to note that this study had a small sample size, which is a limitation. However, the very large effect size (*d* = 1.05) observed between baseline pre-exercise FMD and postexercise FMD reinforced our findings, since effect size calculations do not take sample size into consideration [26]. In addition, of 11 participants in this study, 3 participants were female, which might have influenced the FMD response after exercise. However, no significant gender effect was observed.

## 5. Conclusions

The findings from the present study demonstrated that a single resistance exercise session performed at high intensity (maximal repetition) reduced FMD response in resistance-trained individuals. Given that resistance exercise programs vary widely in terms of variables (i.e., volume, intensity), these findings are important to better comprehend the effect of a single resistance exercise session on FMD, which may be advantageous for the prescription of appropriate exercise protocols. However, long-term resistance exercise promotes a beneficial effect on vascular function and its practice should still be recommended.

## Figures and Tables

**Table 1:** Baseline characteristics of the participants.

**Demographics**	**Mean ± SD **
Number of participants (female)	11 (3)
Age (years)	24.36 ± 5.14
Weight (kg)	76.23 ± 8.10
Height (cm)	171.27 ± 7.50
BMI (kg/m^2^)	26.03 ± 2.74
**Biochemistry**	
Glucose (mg/dL)	86.28 ± 8.91
Total cholesterol (mg/dL)	158.18 ± 40.5
LDL cholesterol (mg/dL)	98.09 ± 47.03
HDL cholesterol (mg/dL)	38.66 ± 11.81
Triglycerides (mg/dL)	108.66 ± 39.39
**Clinical measures**	
SBP (mm Hg)	118.00 ± 11.06
DBP (mm Hg)	78.27 ± 6.66

BMI = body mass index; LDL = low-density lipoprotein; HDL = high-density lipoprotein; SBP = systolic blood pressure; DBP = diastolic blood pressure. Values are mean ± standard deviation (SD).
